# Extraction of water bodies from high-resolution remote sensing imagery based on a deep semantic segmentation network

**DOI:** 10.1038/s41598-024-65430-5

**Published:** 2024-06-25

**Authors:** Dechao Sun, Guang Gao, Lijun Huang, Yunpeng Liu, Dongquan Liu

**Affiliations:** 1 College of Digital Technology and Engineering, Ningbo University of Finance & Economics, Ningbo, China; 2 Popsmart Technology (Zhejiang) Co., Ltd., Ningbo, China; 3 Ningbo Foreign Economy & Trade Information Center, Ningbo, China; 4 Ningbo University of Technology, Ningbo, China; 5 Ninghai First People’s Hospital, Ningbo, China

**Keywords:** Water bodies, Deep learning, Remote sensing imagery, Atrous spatial pyramid pooling, Computer science, Electrical and electronic engineering, Urban ecology

## Abstract

The precise delineation of urban aquatic features is of paramount importance in scrutinizing water resources, monitoring floods, and devising water management strategies. Addressing the challenge of indistinct boundaries and the erroneous classification of shadowed regions as water in high-resolution remote sensing imagery, we introduce WaterDeep, which is a novel deep learning framework inspired by the DeepLabV3 + architecture and an innovative fusion mechanism for high- and low-level features. This methodology first creates a comprehensive dataset of high-resolution remote sensing images, then progresses through the Xception baseline network for low-level feature extraction, and harnesses densely connected Atrous Spatial Pyramid Pooling (ASPP) modules to assimilate multi-scale data into sophisticated high-level features. Subsequently, the network decoder amalgamates the elemental and intricate features and applies dual-line interpolation to the amalgamated dataset to extract aqueous formations from the remote images. Experimental evidence substantiates that WaterDeep outperforms its existing deep learning counterparts, achieving a stellar overall accuracy of 99.284%, FWIoU of 95.58%, precision of 97.562%, recall of 95.486%, and F1 score of 96.513%. It also excels in the precise demarcation of edges and the discernment of shadows cast by urban infrastructure. The superior efficacy of the proposed method in differentiating water bodies in complex urban environments has significant practical applications in real-world contexts.

## Introduction

Water is an important component of the ecosystem. Rapid urbanization, population growth, environmental degradation, climate change, and other factors have resulted in a yearly decrease in water resources, particularly in urban surface waters. Therefore, the timely and effective detection of surface water, delineation of its extent, and real-time monitoring of its dynamics are critical for water resource investigations, flood detection, and water conservation planning^1,2^.

In recent years, advancements in remote sensing technology and enhancements in sensor performance have led to continuous improvements in the spatial resolution of remote sensing images. This progression has enriched the details contained within these images and provided substantial data support for the accurate and rapid extraction of surface water^3,4^. However, the diverse attributes and details of water bodies in high-resolution images add complexity to the capture and utilization of their effective characteristics in high-resolution remote sensing images. Traditional methods for extracting rivers from remote sensing images encounter challenges such as the artificial determination of thresholds, limited automation, and subpar real-time performance, thus making it difficult to promptly delineate regional water bodies^5–8^.

Deep learning methods have strong feature expression abilities and strong applicability in remote sensing image information extraction^9^. The extraction of water information from remote sensing images has become a popular research topic in academia and industry. However, small rivers and small paddy fields may be present in the water body, and river bends are complex; therefore, the design of an efficient deep learning network structure that can accurately recognize rivers in complex environments is still an important issue. In this study, a novel network called WaterDeep is proposed based on DeepLabV3+^10^. The proposed WaterDeep method reconstructs the ASPP module in the network by means of dense connection to expand the range of the network receptive field; at the same time, the low-level features of the backbone network and high-level features of the decoder are fused at multiple levels to enhance its extraction ability for water details.

The major contributions of this study include:

1. A new Atrous Spatial Pyramid Pooling module is designed with dense connections to enhance the ability of the network to accommodate the varying receptive fields required for complex water-scene recognition. In the designed module, the dilation rates increase gradually across layers. The upper-level dilated convolutions leverage the results of the lower-level dilated convolutions, enabling the acquisition of a denser range of receptive fields and denser pixel extraction.
2. A new decoder module is designed that leverages feature fusion. This module fuses low- and high-level feature maps, enriching the image detail and refining the edges in semantic segmentation, thereby improving the delineation of water features.

The remainder of this paper is organized as follows: Section "Related studies" discusses related studies, Section "Materials and methods" details the improved algorithm, Section "Experiments and results" presents the experiments and results, Section "Discussion" presents a discussion of the results, and Section "Conclusions" concludes the work.

## Related studies

At present, methods for the automatic extraction of water bodies from remote sensing images can mainly be divided into threshold methods^11,12^, classifier methods^13,14^, and deep learning methods. Threshold methods analyze the spectral characteristic curve of water, select a single band or different band combinations in the image to construct an appropriate water extraction model, and define a certain threshold for the model to separate water from other ground objects and extract water information. The main threshold methods include the ratio method, band threshold method, and water index model method. The ratio and band threshold methods have simple principles and fast extraction speeds, but they are often unable to distinguish shadows, vegetation, and other ground objects close to the reflectivity of the water body, thus resulting in low extraction accuracy for water body information. The water body index model method^15–17^ can accurately distinguish water bodies and vegetation information, but the extraction accuracy of rivers is difficult to control.

Classifier methods design a classifier for the adopted image features, divide the image into categories based on certain algorithm rules, and then extract water information from the image. They are mainly divided into decision tree, support vector machine (SVM), and object-oriented methods. The decision tree method^8^ extracts feature information according to certain classification principles through a comprehensive induction and experience summary of different data. Although its extraction accuracy is high, its rules are difficult to determine. The SVM method has the characteristic of small sample training and performs well for high-dimensional pattern recognition^18^. However, in practical applications, it is difficult to find appropriate model parameters because of the traditional grid search, which affects its recognition accuracy for rivers. The object-oriented method^9^ divides the image into several regions or objects, takes the object as the basic unit for image classification, and uses the characteristics of the spectrum, texture, shape, and topology to classify ground objects. However, different segmentation scales often result in varying extraction accuracies and cumbersome operation. The classifier extraction method can remove the influence of shadows and buildings to a certain extent; however, its feature extraction and classifier design depend on expert knowledge, and different regions and images are not universal.

The water body extraction method based on deep learning models is a new method developed in recent years. It performs feature extraction and self-learning of water samples from remote sensing images using an artificial neural network to achieve automatic discrimination and prediction of water bodies. Deep learning models can extract and fit the features of high-dimensional image data more effectively. Therefore, they have strong applicability in information extraction from high-resolution remote sensing images.

In recent years, the development of fully convolutional networks has led to significant progress in deep learning. Fully convolutional networks provide a new solution for river surface extraction from remote sensing images^10^. Many excellent semantic segmentation algorithms have been proposed. SegNet records the reserved space location information in the pool layer, which further improves the segmentation accuracy^19^. UNet adopts a U-shaped structure, which is more conducive to transmitting shallow information to deep layers^20,21^. Wang et al.^22^ used hybrid dilated convolution instead of a dilated convolution to expand the receptive field. Compared with other traditional methods, a fully convolutional neural network has more advantages for river extraction^23,24^. Yu et al.^25^ applied a convolutional network for surface river extraction, and the effect was clearly better than that of the traditional water extraction method^26–28^. Wang et al.^29^ proposed an end-to-end trainable multi-scale lake water extraction network (MSLWENEt) that could effectively extract the water bodies of small lakes, thus solving the problems of large intra-class variance and small inter-class variance in lake water bodies. Liu et al. ^30^ proposed a novel SFnet-DA network based on domain adaptation (DA) by embedding a selective self-attention (SSA) mechanism and multi-scale feature fusion (MFF) module. Benefitting from the SSA mechanism, SFnet-DA could selectively extract water bodies of different sizes and alleviate the influence of noise.

The above methods can effectively extract water bodies but are still susceptible to problems such as complex image backgrounds, difficulty distinguishing adjacent objects with similar colors, significant shadow noise, and blurred edge contours in water body extraction. In response to these issues, this study improves and optimizes the DeepLabV3+ network by proposing a novel network called WaterDeep. This method reconstructs the ASPP module, thereby expanding the discrimination between adjacent objects of similar colors. Second, in the encoder section, shallow and deep features are fused to solve the problem of segmentation inaccuracy caused by different types of water bodies and large differences in shape and size, thus enhancing the clarity of the water body edge segmentation.

## Materials and methods

**Figure 1:**
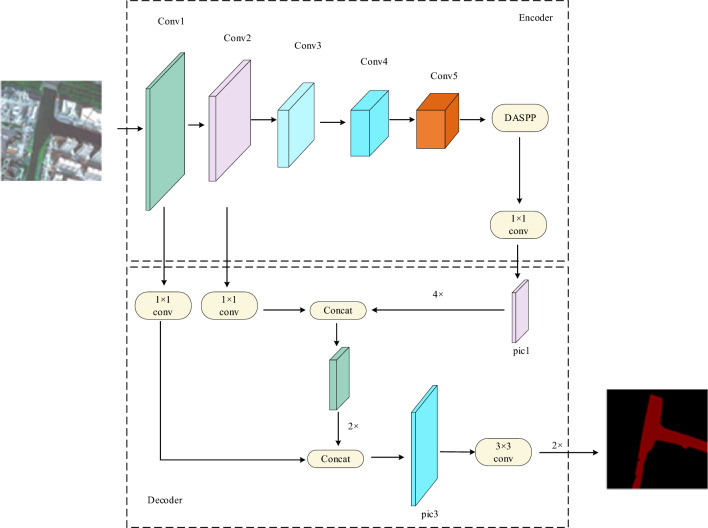
Overall structure of the WaterDeep network.

In this study, the encoder–decoder structure of the classical semantic segmentation DeepLabV3+ network is used to extract water information from remote sensing images. The overall structure of the system is shown in Fig. 1. The encoder module is composed of an improved Xception module and DASPP module. The encoder process is the feature down-sampling process of the image, and its input is a remote sensing image of 512 × 512 pixels. Shallow features are extracted through the Xception network, and then multi-scale information is obtained through the densely connected DASPP module to aggregate global features. After the encoder process is completed, the remote sensing image can be extracted to a size of 32 × 32 × 2048, which is a highly abstract feature map. The decoding process is a feature-recovery process. Specifically, the highly abstract feature map output by the encoder is bilinear up-sampled, and the shallow features of different resolutions corresponding to the Xception network are subjected to a 1 × 1 convolution. The shallow and deep features are then fused; finally, the multi-scale features are interpolated to extract water information from the remote sensing images.

### Improved Xception module

**Figure 2:**
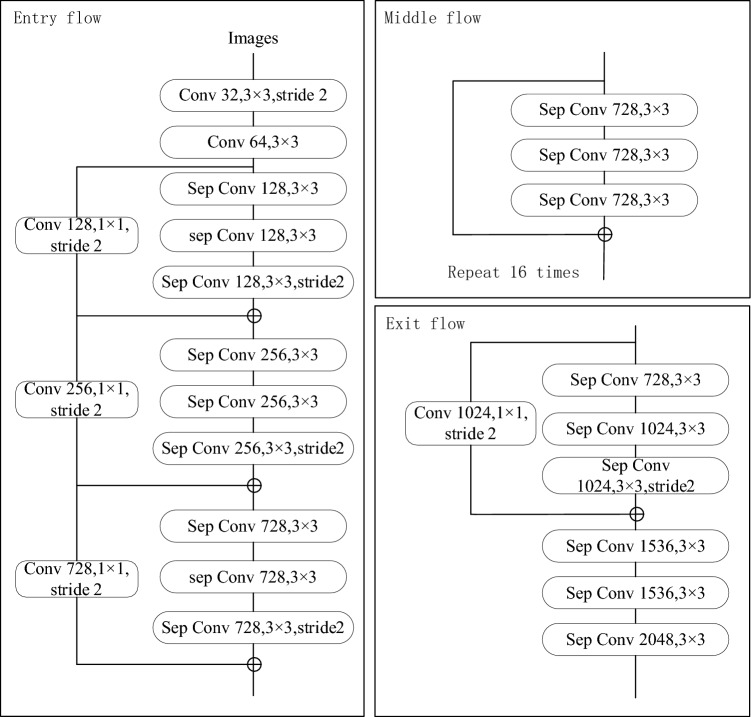
Schematic diagram of the improved Xception network structure.

Xception is used as the backbone feature-extraction network. Xception is a network proposed by Google in 2017. As shown in Fig. 2, the Xception network framework is divided into three parts: entry, middle, and exit flows. The entry flow includes two convolutions and three residual modules, which are used to down-sample the input image to reduce the spatial size. The middle stream includes 16 residual blocks, which are used to continuously learn the correlation and optimize the features. The exit stream includes two residual modules, which are used to sort the features to obtain a rough score map.

Based on the size of the output characteristic map, the Xception network is divided into five parts: conv1 (256 × 256), conv2 (128 × 128), conv3 (64 × 64), conv4 (32 × 32), and conv5 (32 × 32), as listed in Table 1. The size of the input image is 512 × 512 pixels. After extracting the information in conv1, conv2, conv3, and conv4 with a step size of 2, and conv5 with a step size of 1, the output size is 32 × 32. To extract water features more effectively, this study improves the Xception network: the number of intermediate flow layers is increased, and the linear stacking of deep separable convolution layers is increased from eight to 16 times. Moreover, the original pooling layer is changed to a depth-separable convolution with a stride of 2, additional Relu layers and normalization operations are added in each 3 × 3 after deep convolution, and the full connection layer and logistic regression layer of the Xception network are removed to ensure the characteristics of the spatial dimension of the classification network output.

**Table 1:** Xception composition result.

Stage	Output dimension	Corresponding part of Xception	
conv1	256 × 256 × 64	Entry flow	3 × 3, 32, stride = 2
			3 × 3, 64
conv2	128 × 128 × 128	Entry flow	Sep conv 3 × 3, 128
			Sep conv 3 × 3, 128
			Sep conv 3 × 3, 128 stride = 2
conv3	64 × 64 × 256	Entry flow	Sep conv 3 × 3, 256
			Sep conv 3 × 3, 256
			Sep conv 3 × 3, 256 stride = 2
conv4	32 × 32 × 728	Entry flow	Step conv 3 × 3, 728
			Step conv 3 × 3, 728
			Step conv 3 × 3, 728 stride = 2
		Middle flow	Step conv 3 × 3, 728
			Step conv 3 × 3, 728 × 16
			Step conv 3 × 3, 728 stride = 2
conv5	32 × 32 × 2048	Exit flow	Step conv 3 × 3, 728
			Step conv 3 × 3, 1024
			Step conv 3 × 3, 1024 stride = 2
		Exit flow	Step conv 3 × 3, 1536
			Step conv 3 × 3, 1536
			Step conv 3 × 3, 2048 stride = 2

### DASPP module

**Figure 3:**
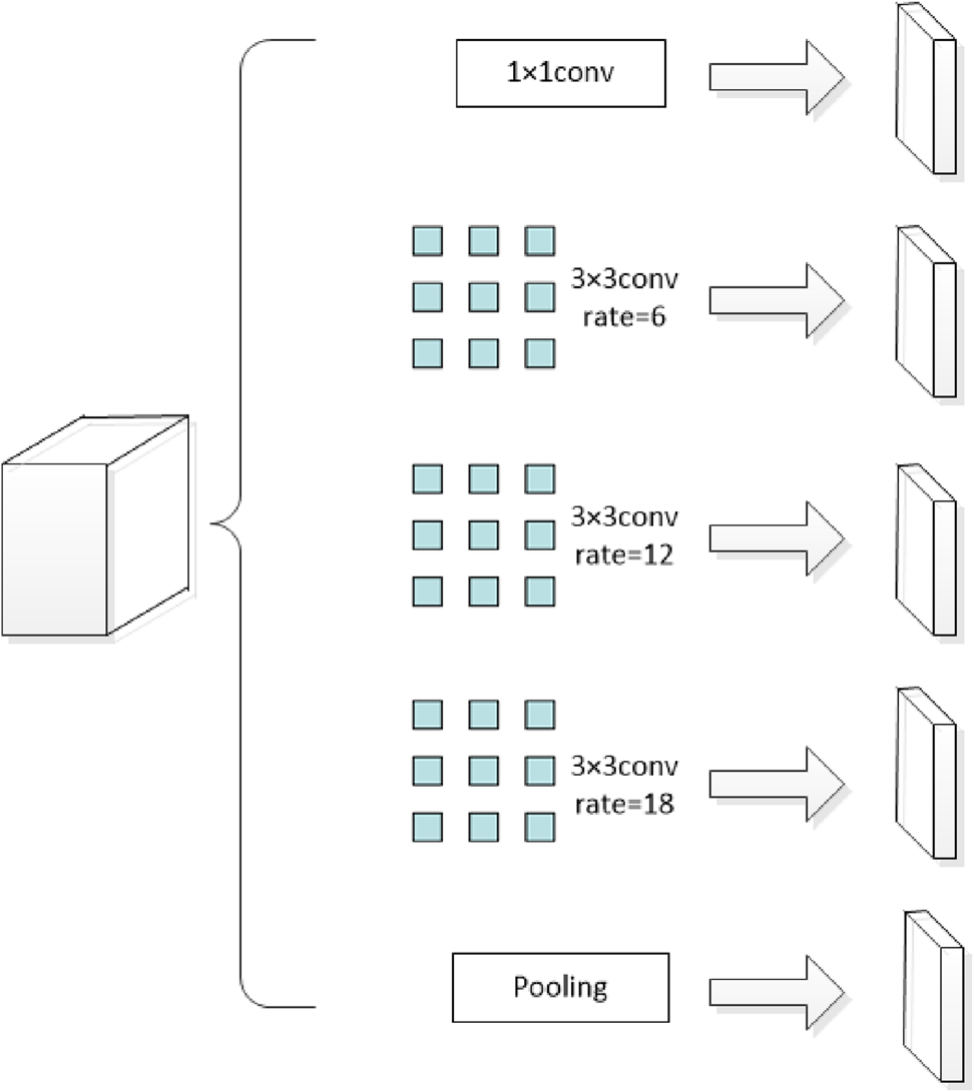
ASPP module structure diagram.

**Figure 4:**
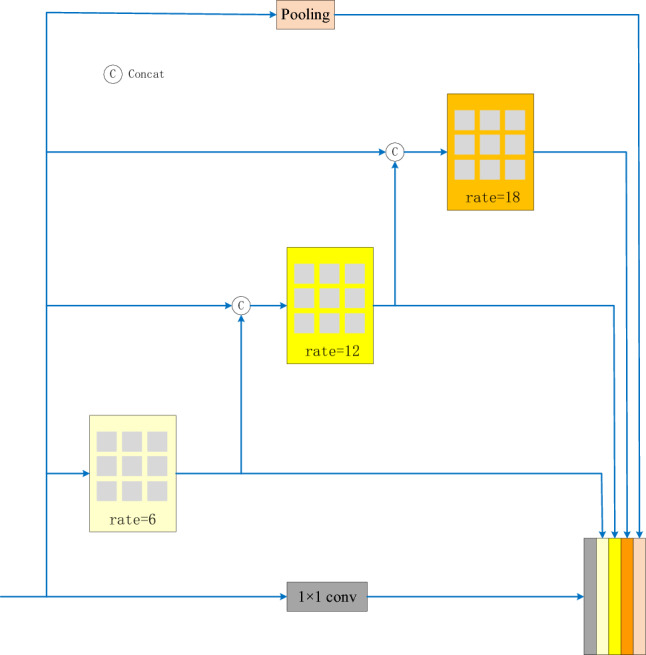
DASPP module structure.

The ASPP module of the DeepLabV3+ network has one 1 × 1 convolution and three 3 × 3 dilation convolutions with dilation rates of 6, 12, and 18. The structure of the ASPP is shown in Fig. 3. The DeepLabV3+ network takes the feature map extracted from the Xception base network as the input to the ASPP module; after parallel convolution calculations of different dilation rates, it is fused to cover the receptive field on a large scale. However, during the process of water body information recognition, rivers are constantly changing, and the water surface and surrounding scenery are also very uncertain. When the surrounding environment changes significantly, the ASPP module cannot adapt well to the changes in the target. Therefore, this study reconstructs the ASPP in the original network into a DASPP module in the form of a dense connection, expands the range of its receptive field, and obtains denser pixel extraction to adapt to the extraction of complex and changeable water body information, especially the extraction of small water bodies. The DASPP module is shown in Fig. 4.

**Figure 5:**
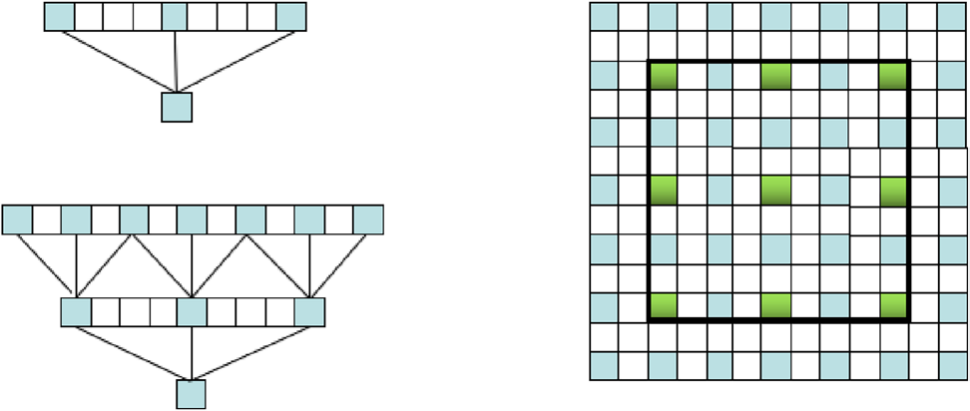
DASPP module pixel sampling diagram.

Compared with the ASPP module in the original network, the DASPP module can obtain multi-scale information and enable more pixels to participate in the calculation process. In fact, because of the “zero filling” operation of dilation convolution, the pixel samples of hole convolution are relatively sparse. In the 1D dilation convolution layer, the convolution kernel receptive field with a hole rate of four is nine, and only three pixels are sampled and calculated. If a dense connection method is adopted, seven pixels will be sampled and calculated. In the 2D image, convolution with an excessively high dilation rate leads to the loss of a large amount of pixel information. If a convolution layer with a dilation rate of two is placed under the convolution layer with a hole rate of four, and a densely connected convolution layer is used, 49 pixel points are involved in the calculation. Compared with the nine pixel points used in the single-layer convolution, the densely connected convolution layer is more conducive to obtaining more calculation information and expanding the amount of pixel information collected, as shown in Fig. 5.

In addition, the DASPP module expands the range of the receptive field. In the Deeplabv3 network, when the size of the dilation convolution kernel is *K* and the dilation rate is *r*, the size of the receptive field, ${R}_{r}^{k}$, that can be provided is given by Eq. (1).

1 $${R}_{r}^{k}=\left(r-1\right)\times \left(k-1\right)+k$$

If the two-layer dilation convolutions are concatenated, the size of the receptive field can be obtained as follows:

2 $$R=R1+R2-1,$$

where *R*1 and *R*2 are the receptive fields provided by the two-layer dilation convolutions.

Therefore, the maximum receptive field obtained using the ASPP module in the DeepLabV3+ network is calculated using Eq. (3).

3 $$R=\text{max}\left({R}_{6 }^{3}{,R}_{12 }^{3}{,R}_{18 }^{3}\right)={R}_{18 }^{3}=\left(18-1\right)\times \left(3-1\right)+3=37$$

In the improved DASPP module, the receptive field can be obtained using Eq. (4).

4 $$R={R}_{6 }^{3}{+R}_{12 }^{3}{+R}_{18 }^{3}-2=73$$

### Feature fusion module

In the process of gradually recovering the image size, the DeepLabV3+ network decoder module uses multiple large up-samplings to ignore the semantic information of the feature map, which is not conducive to the formation of a high-level feature map. A large number of image details are ignored in the large-scale up-sampling process, which leads to blurring of the extracted water edge contour. Therefore, in the decoder module of the network, a combination of low-level and high-level feature maps is used to endow the up-sampled feature map with more image details, refine the edge of the semantic segmentation image, and enhance the semantic segmentation effect of the water body.

**Figure 6:**
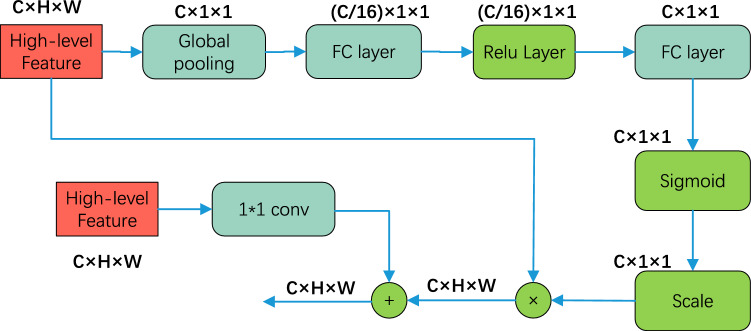
Schematic diagram of high-low level feature fusion.

Therefore, a fusion algorithm is proposed, which acts on two adjacent features and gradually merges from high to low levels. The network structure of the fusion algorithm is shown in Fig. 6. It can be divided into two main parts: compression and activation. First, the feature map is compressed by Global Average Pooling, which is expressed as follows:

5 $${F}_{SQ}\left({x}_{c}\right)=\frac{1}{H\times W}{\sum }_{i=1}^{H}{\sum }_{j=1}^{W}{x}_{c}\left(i,j\right),$$

where *F* represents the feature map with size *H* × *W* × *C*, the total number of channels is *c*, and *x* is the two-dimensional matrix with channel *c* in the feature map, which is then activated. The expression is:

6 $${S}_{C}=\delta \left({w}_{2}\sigma \left({w}_{1}{z}_{c}\right)\right),$$

where the dimension is reduced by the Fully Connected(FC) layer with weight ${w}_{1}$, and then the dimension is increased by the FC layer with weight ${w}_{2}$. In the middle, the Relu function, $\sigma$, is used to activate, and then the output is normalized by the Sigmoid function, $\delta$. Finally, the normalized data is used as a scale factor to multiply the c channels of input high-level feature, and after being fused with low-level feature, it will be used as the input for the next level. As shown in Fig. 1, The fusion algorithm first fuses the high-level features pic1 output by DASPP and high-level features output by conv2 into fea1, and then fuses the features output by fea1 and conv1 into pic3; finally, pic3 is up-sampled to achieve the final segmentation image. The principle of the feature fusion module is to enhance important channel features and weaken unimportant features by different scale value, thus making the extracted features more directional.

## Experiments and results

### Datasets

**Figure 7:**
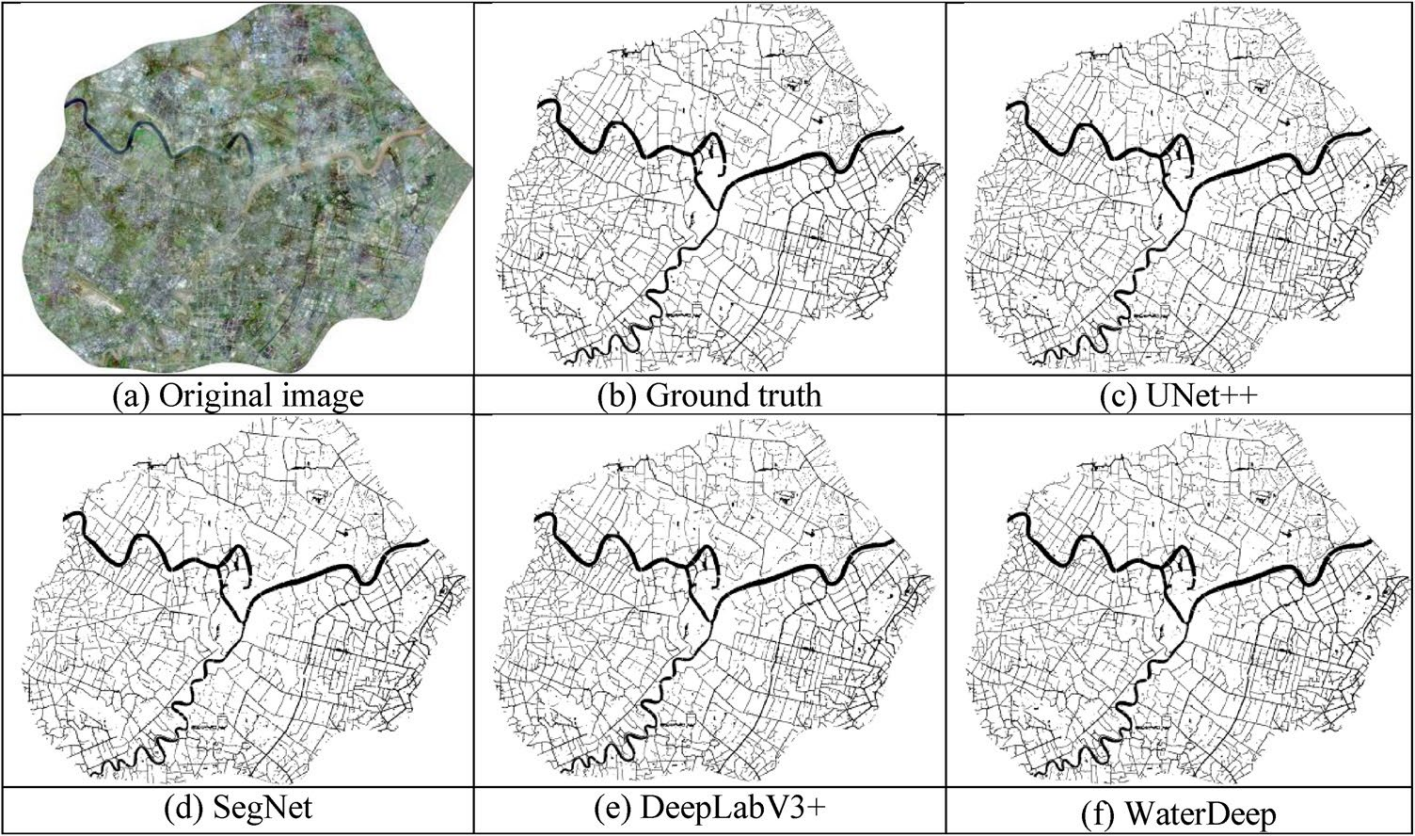
Overall water extraction results using UNet +  + , SegNet, DeepLabV3 + , and the proposed WaterDeep methods. The map is designed in qgis V3.16.16 software available at qgis website (https://download.qgis.org/downloads/).

The remote sensing image data used in this study are the 0.5 m true color data after panchromatic and multispectral fusion collected by the GeoEye-1 satellite in Ningbo City, Zhejiang Province, China, covering an area of approximately 420 km^2^. The water distribution in the experimental area is extremely complex. Three major rivers (the Yao River, Fenghua River, and Yong River) run through the city, accompanied by smaller rivers as dense as cobwebs. Field ponds, irrigation canals, and other surface covers are also present. With the assistance of basic geographic information data, the real label image corresponding to the water bodies was obtained by manual visual interpretation, and the data were then cut into 512 × 512 pieces; the training, verification, and test sets were divided according to the ratio of 6:2:2. The image-labeling process is illustrated in Fig. 7. Figure 7a shows the original image, and Fig. 7b shows the corresponding image label diagram. To improve the generalization ability of the network, the training set was expanded to 1500 pieces using operations such as image flipping and enhancement to improve the diversity of the data.

### Experimental environment and evaluation method

The computer processor used for these experiments was an Intel I7 12700KF, with an NVIDIA RTX 3080TI 12GB graphics card and 64GB of RAM. The operating system was Ubuntu 20.04, the deep learning framework was PYTORCH V1.12, and the parallel computing framework was CUDA 11.3. The initial learning rate was set to 0.0005, the batch size was 32, and the training iteration epoch was 100 rounds.

The overall accuracy (OA), frequency-weighted intersection over union (FWIoU), precision, recall, and F1 score are used to assess the quantitative performance of the experiments. The OA is calculated as follows:

7 $$OA=\left({p}_{11}+{p}_{22})/({p}_{11}+{p}_{22}+{p}_{12}+{p}_{21}\right).$$

*FWIoU* is calculated as follows:

8 $$FWIoU=\frac{1}{\sum_{i=1}^{2}\sum_{j=1}^{2}{p}_{ij}}\sum_{i=1}^{2}\frac{{p}_{ii}\sum_{j=1}^{2}{p}_{ij}}{\sum_{j=1}^{2}{p}_{ij}+\sum_{j=1}^{2}{p}_{ji}-{p}_{ii}},$$

where ${p}_{11}$ is the number of correctly extracted pixels, ${p}_{22}$ is the number of correctly assigned non-water body pixels, ${p}_{12}$ is the number of pixels that detect water as non-water bodies, and ${p}_{21}$ is the number of pixels that detect non-water bodies as water bodies.

*Precision* is the ratio of the correctly detected water body pixels to all of the detected water body pixels. The *Recall* rate is the ratio of the correctly detected water body pixels to all of the water body pixels. The *F1* score is an important evaluation standard for measuring the accuracy of secondary classifications. It also considers the *Precision* and *Recall* rates of the classification results. These indexes are defined as follows:

9 $$Precision=\frac{TP}{TP+FP} , Recall=\frac{TP}{TP+FN} , F1= \frac{2(Precision\times Recall)}{(Precision+Recall)},$$

where *TP*, *FN*, *FP*, and *TN* are the pixels categorized by comparing the extracted water pixels with the ground truth reference. TP: true positives, i.e., the number of correctly extracted pixels; FN: false negatives, i.e., the number of water pixels not extracted; FP: false positives, i.e., the number of incorrectly extracted pixels; TN: true negatives, i.e., the number of non-water body pixels that were correctly extracted.

### Experimental results

The training, verification, and test sets used 512 × 512 images. To observe the overall effect of water body extraction, we stitched the extracted feature maps according to the corresponding positions and restored them to the original image size of 57342 × 46824 pixels. The extracted water body information results for the four algorithms are shown in Fig. 7. The black extraction results represent water bodies. Figure 7a–f show the original image, ground truth, UNet++, SegNet, Deeplabv3+, and proposed WaterDeep results. Overall, because of the strong feature representation ability of deep learning, the main water body structures extracted using the four methods are complete, the water body contours are clear, and the area is complete.

## Discussion

The performance of the water body extraction algorithm is evaluated through five evaluation metrics and visual results. According to the evaluation results in Table 2, the OA, FWIoU, precision, recall and F1 values obtained with the proposed water extraction algorithm are 99.284%, 95.58%, 97.562%, 95.486%, and 96.513%, respectively, which are better than the results obtained with the other three methods.

**Table 2:** Comparison of the results of the proposed network with other networks based on the OA, FWIoU, precision, recall and F1 score.

	OA	FWIoU	Precision	Recall	F1
UNet + +	99.025%	93.507%	95.822%	92.772%	94.272%
SegNet	98.744%	92.877%	96.579%	89.503%	92.906%
DeepLabV3 + +	98.929%	94.539%	96.717%	92.989%	94.816%
WaterDeep	99.284%	95.58%	97.562%	95.486%	96.513%

Due to the large-scale resizing of the image sizes in Fig. 7c–f , the results cannot reflect the subtle differences in water body extraction under conditions of complex backgrounds and similar object colors. To further analyze the effectiveness of the algorithm, the effects of the algorithm are further compared and discussed in this section in terms of four additional aspects: shadows, small water detection, edges, and integrity.

### Ability of different methods to distinguish similar colours

**Figure 8:**
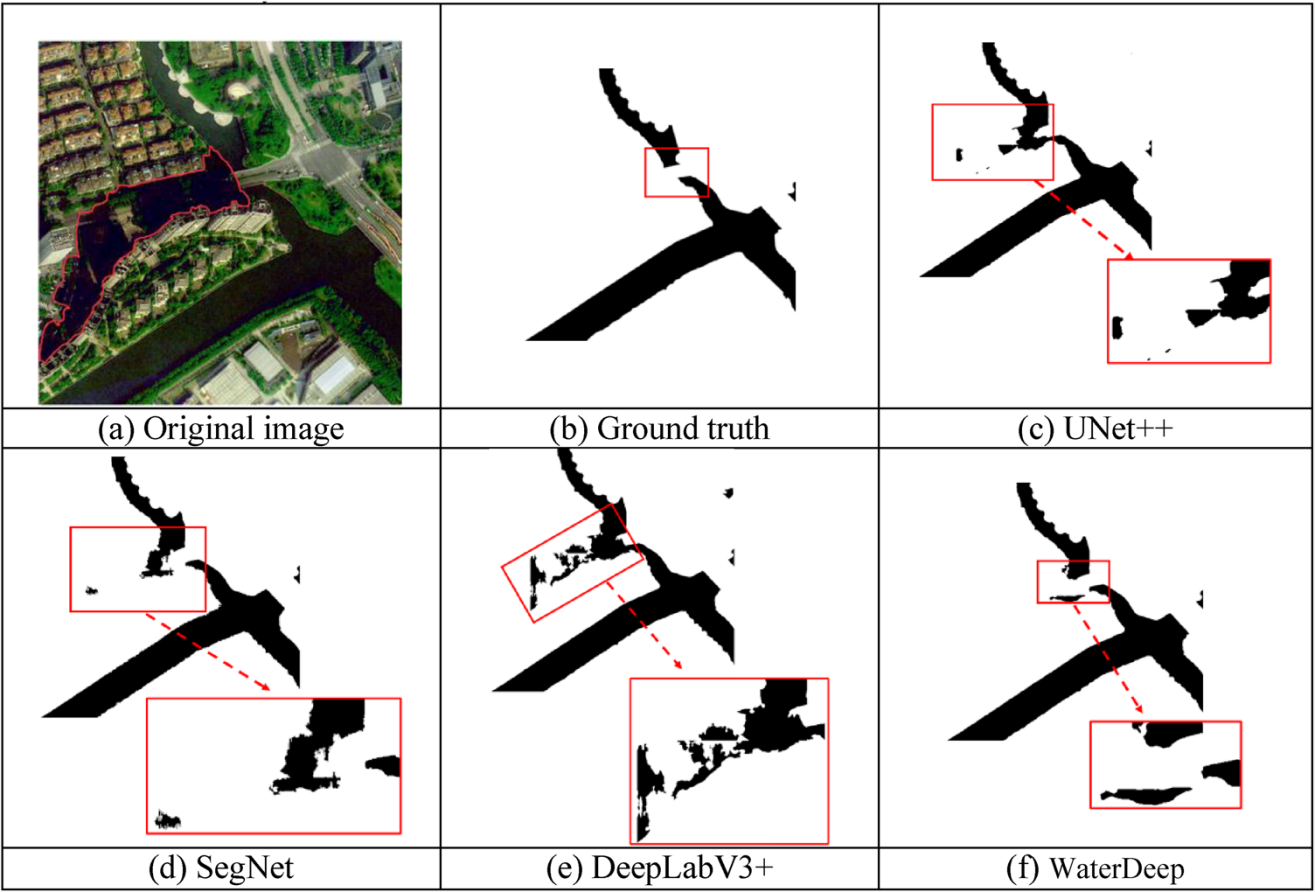
Ability of UNet +  + , SegNet, DeepLabV3 + , and the proposed WaterDeep methods to distinguish shadows. The Figure is drawn by the Pillow library 10.30 in Python 3.8 available at (https://pypi.org/project/pillow/#files).

The shadow areas and water bodies belong to low-reflectance objects, with their colour gray values often very close to each other, which makes it easy to confuse shadows and water bodies when extracting water body information. When algorithms attempt to differentiate between water bodies and shadows, this similarity often results in identification errors, thereby affecting the accuracy and reliability of the extraction results. To validate the performance of our improved method, we compared it with several classical algorithms, including U-net++, SegNet, and DeepLabV3+. These algorithms have extensive applications and excellent performance in image segmentation and geographic information extraction. Images including shadows and water bodies were selected as experimental images, and the red circle in Fig. 8a indicates the shaded area, while the other black parts represent water bodies.

Figure 8c–f shows the water extraction results using U-net++, SegNet, DeepLabV3+, and the proposed method. By comparing the extraction results of various methods, it can be observed that although all four methods can identify water body areas to some extent, there are significant differences in their performance when dealing with shadows. Specifically, the method based on DeepLabV3+ incorrectly identifies most of the shadow areas as water bodies in Figure 8e, evidently due to the similarity in grayscale values between shadows and water bodies. Additionally, in Figures 8c and d, some obvious identification errors can be observed, further indicating the interference of shadows in water body extraction.

Fig. 8f shows the results achieved by our improved method, which basically achieves the effect of the ground truth, as shown in Fig.8b. Our WaterDeep method accomplishes this through the DASPP module, gradually increasing the network's dilation rate. The upper-layer dilation convolution utilizes the results of the lower-layer dilation convolution to obtain a denser receptive field range and more densely extracted pixels. Therefore, it not only accurately distinguishes between water bodies and shadows but also preserves more detailed information, making the extraction results closer to reality.

### Extraction effect for small water bodies

**Figure 9:**
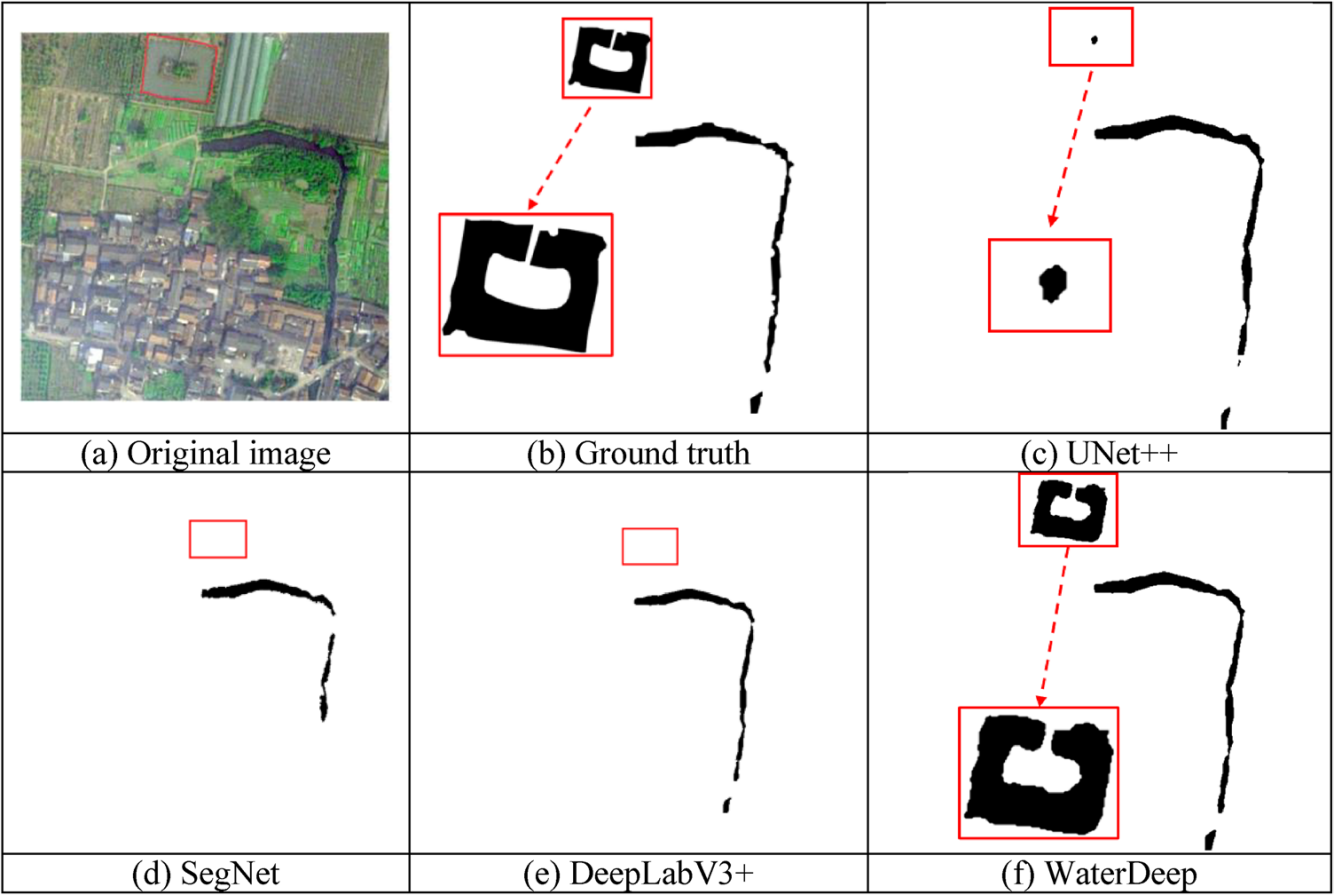
Extraction of a small water body using UNet +  + , SegNet, DeepLabV3 + , and the proposed WaterDeep methods. The Figure is drawn by the Pillow library 10.30 in Python 3.8 available at (https://pypi.org/project/pillow/#files).

Small bodies of water often occupy a very small pixel range in remote sensing images or ground data, and their characteristic information is easily concealed by the complex background surrounding them. Due to the limitations of dataset size and model structure, convolutional calculations may overlook certain features of small bodies of water, making their effective identification difficult. The red area in Fig. 9a represents a pond, which is a small body of water, while the background contains information about ground objects. Fig. 9b shows the annotation information, and the black patch above it represents the true segmentation result corresponding to the pond. Figures 9c–f demonstrate the results of extracting pond water using UNet++, SegNet, DeepLabV3+, and the proposed method, respectively. The method based on SegNet in Fig. 9d and the method based on DeepLabV3+ in Fig. 9e focus on overall structural segmentation, lacking sufficient capture of local details, thus they are ineffective in identifying small bodies of water such as ponds.

The method based on UNet++ in Figure 9c is an improved version of the U-Net structure, enhancing the ability of feature extraction by introducing more skip connections and deeper network layers. However, when dealing with small bodies of water, there may still be limitations due to the complexity of its network structure, resulting in the recognition of only partial water areas. In contrast, our proposed WaterDeep method achieves enhanced image segmentation by incorporating low-level features from the Xception encoder in the decoding module and fusing them with high-level feature maps, and thus the proposed method can effectively identify small water bodies such as ponds.

### Comparison of the water body edge recognition effect and extraction integrity

**Figure 10:**
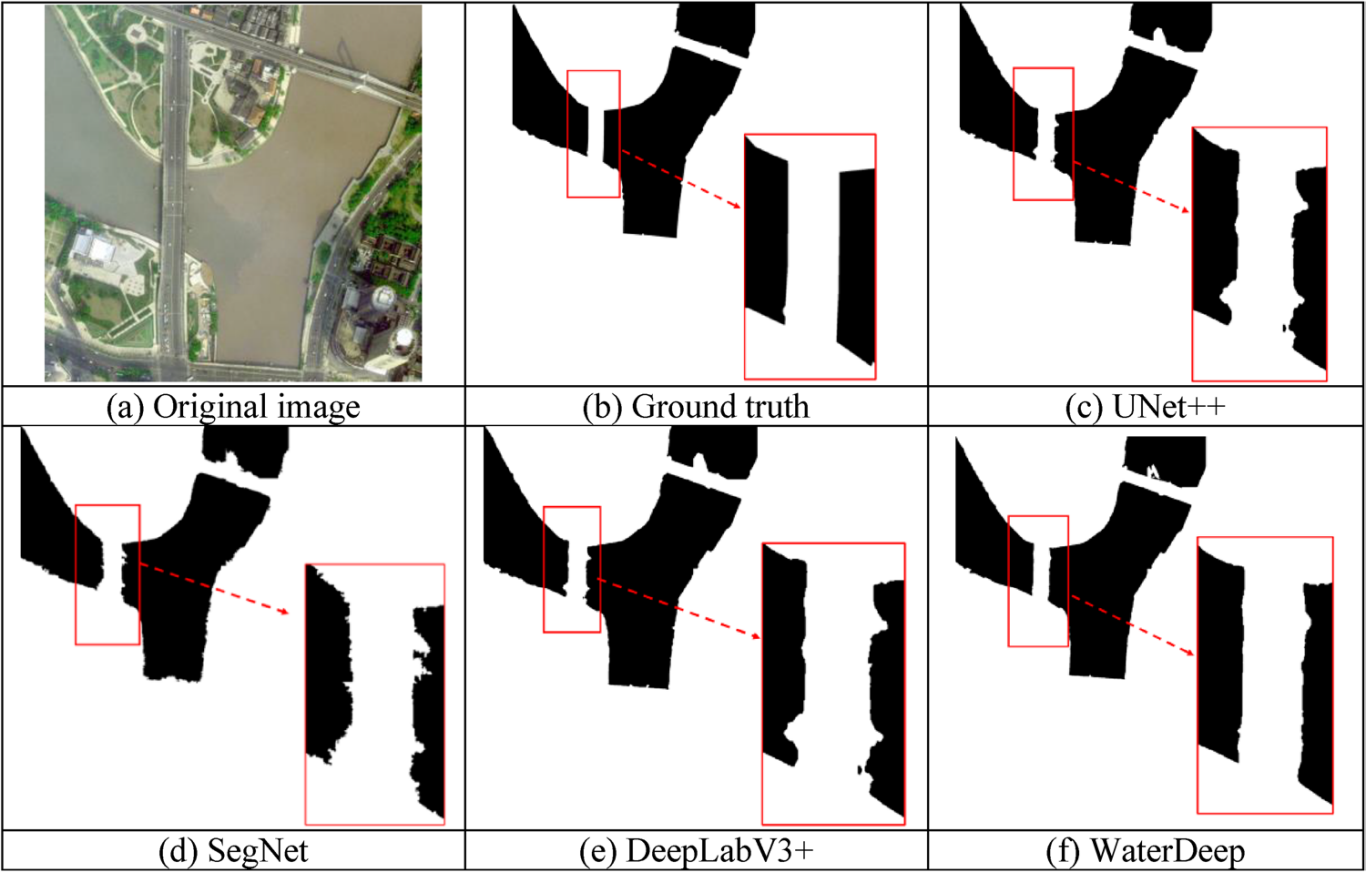
Comparison of the water body edge recognition effect using UNet +  + , SegNet, DeepLabV3 + , and the proposed WaterDeep methods. The Figure is drawn by the Pillow library 10.30 in Python 3.8 available at (https://pypi.org/project/pillow/#files).

The precision of water body edge segmentation is an important index for measuring the effect of water body information extraction. This study compares the water body edge extraction information obtained with several methods. Fig. 10a shows the original image containing both the bridge and the water body simultaneously. Figure 10c–f show the semantic segmentation diagrams of the water body information extracted using UNet++, SegNet, DeepLabV3+, and the proposed method. Compared with the real segmentation results in Fig. 10b, it can be seen that the water area extracted by the UNet++, SegNet, and DeepLabV3+ algorithms can effectively recognize the bridge partition, but the edge of the water area is rough. The improved WaterDeep algorithm provides clear water extraction boundary contours in the experimental results (Fig. 10f), which can accurately identify bridges in the water and improve the extraction accuracy of water information.

**Figure 11:**
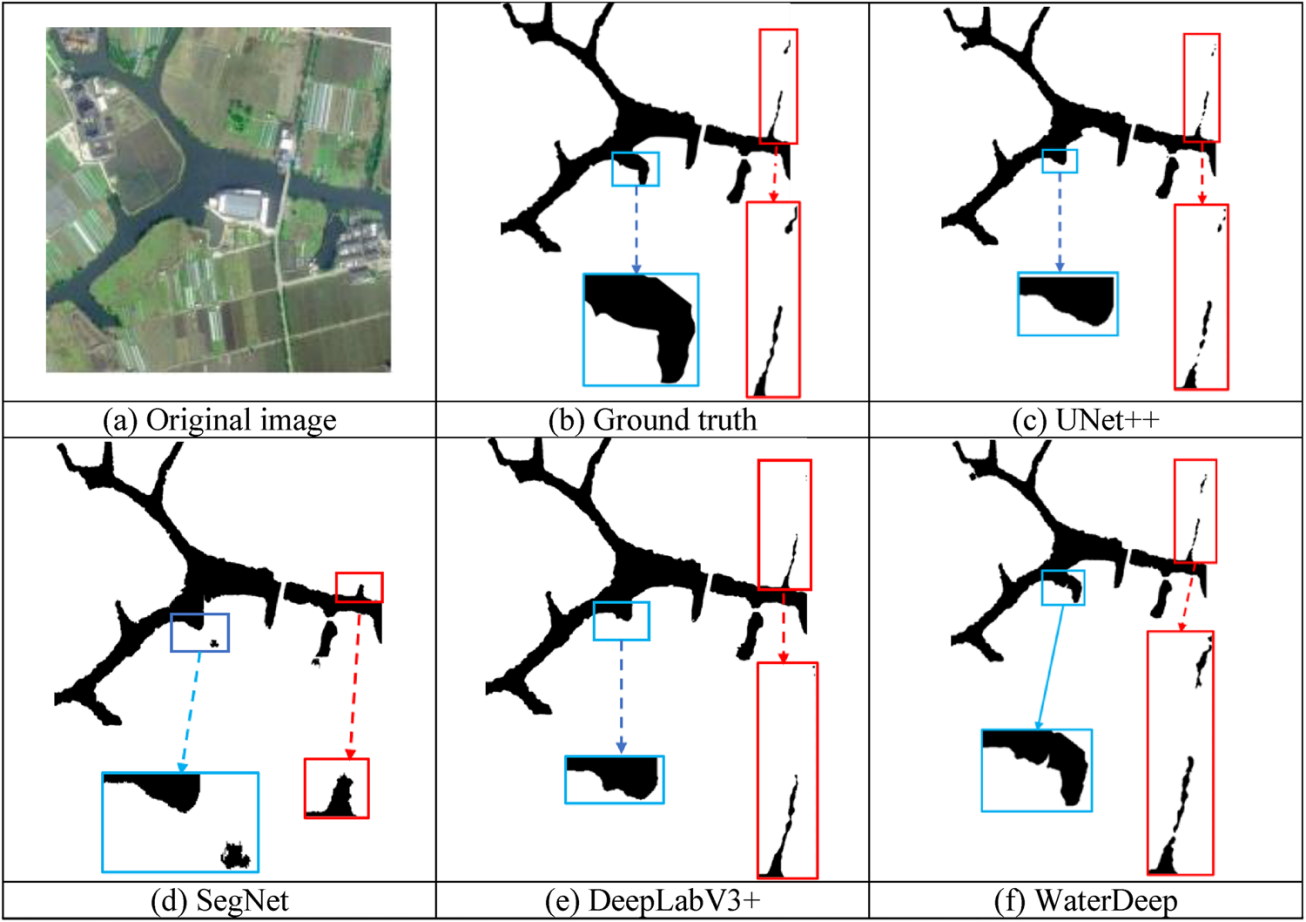
Comparison of the water body extraction integrity using UNet +  + , SegNet, DeepLabV3 + , and the proposed WaterDeep methods. The Figure is drawn by the Pillow library 10.30 in Python 3.8 available at (https://pypi.org/project/pillow/#files).

Surface water bodies include not only large rivers but also narrow water bodies such as streams and ponds. Therefore, this study compares the integrity of the water body extraction information obtained using different methods. Fig. 11a is the original image, which contains many slender black water bodies. Fig. 11c–f show the results extracted using UNet++, SegNet, DeepLabV3+, and the proposed WaterDeep methods. Compared with the true segmentation results in Fig. 11b, the results of the four river extraction algorithms are relatively complete, but there are some subtle differences. The results of the four algorithms for extracting rivers are relatively complete. The UNet++ algorithm recognizes small rivers with discontinuities and end losses, whereas the SegNet algorithm does not recognize small rivers. At the same time, shadows are mistakenly recognized as water bodies. DeepLabV3+ loses some water body information for small rivers. The proposed WaterDeep algorithm has good integrity for water body recognition. Although there are a few interruptions, it basically achieves the effect of the real annotation.

## Conclusions

Water body extraction from remote sensing images suffers from the problem that the obtained water body contour edge is fuzzy and continuous blocks of buildings or vegetation shadows may be recognized as water bodies. To address these problems, this study proposes a water body information extraction algorithm based on a convolutional neural network. The algorithm modifies the Xception backbone network to extract the low-level features of the input image more effectively, and a DASPP module based on dense connections is designed to realize high-level feature extraction with larger semantic information. In the up-sampling stage, the low-level features of conv1 and conv2 and the high-level features of the DASPP output are fused to solve the problem of loss of feature information and finally realize accurate image semantic segmentation. Through a quantitative accuracy evaluation, the OA of this method is 99.284%, the FWIOU is 95.58%, the precision is 97.562%, the recall is 95.486%, and the F1 score is 96.513%, which are better than the results obtained with other methods of the same type. By comparing the experimental results, the improved algorithm can clarify the contour of the water extraction area, and it performs well in distinguishing vegetation, building shadows, extracting small water bodies, and ensuring integrity of the water bodies. Subsequently, various information in remote sensing images can be extracted by adding data set samples and combining multiple extraction models.

## Data Availability

Readers can access our data by sending an email to the corresponding author.
